# Association between the non-high-density lipoprotein cholesterol to high-density lipoprotein cholesterol ratio and carotid plaque: a retrospective cohort study

**DOI:** 10.3389/fnut.2026.1792724

**Published:** 2026-04-28

**Authors:** Jingshan Jiang, Shuang Liu, Yangxuan He, Jiayi Deng, Yunxiang Ming, Yilin Zhu, Shusheng Fang, Yang Liu, Song Leng

**Affiliations:** 1 Health Management Center of the Second Affiliated Hospital of Dalian Medical University, Dalian, Liaoning, China; 2 Department of Health Inspection and Quarantine, School of Life Sciences, Wuchang University of Technology, Wuhan, Hubei, China

**Keywords:** carotid atherosclerosis, carotid plaque, cohort study, NHHR, threshold effect

## Abstract

**Objective:**

To investigate the association between the non-high-density lipoprotein cholesterol to high-density lipoprotein cholesterol ratio (NHHR) and the risk of carotid plaque (CP).

**Methods:**

In this cohort study, 5,985 adults aged ≥18 years without baseline CP were selected from the Dalian Health Management Cohort (2015–2023). Participants were categorized into quartiles according to NHHR levels. Kaplan–Meier curves were used to estimate cumulative CP incidence. Cox proportional hazards models and restricted cubic spline (RCS) analyses were applied to evaluate the association between NHHR and CP risk. Subgroup and sensitivity analyses were conducted to assess the robustness of the findings.

**Results:**

During 11,725.34 person-years of follow-up (mean follow-up: 2.24 years), 780 participants (13.03%) developed CP. Compared with participants in the lowest NHHR quartile, those in the highest quartile had a significantly increased risk of CP (HR = 1.64, 95% CI: 1.26–2.14; *p* < 0.001). When treated as a continuous variable, NHHR remained independently associated with CP risk (HR = 1.11, 95% CI: 1.04–1.18; *p* = 0.001). RCS analysis demonstrated a nonlinear positive association (P for nonlinearity = 0.003), with an inflection point at NHHR = 4.071. Significant interactions were observed for age, sex, and hypertension status (all P for interaction < 0.05), with stronger associations among individuals aged <45 years, females, and normotensive participants. Sensitivity analyses confirmed the robustness of the results.

**Conclusion:**

Elevated NHHR is independently associated with an increased risk of CP, with effect modification by age, sex, and hypertension status. NHHR may serve as a simple and noninvasive biomarker for early risk stratification of CP.

## Background

Carotid plaque (CP), as a manifestation of carotid atherosclerosis (CAS), is associated with an increased risk of cardiovascular diseases (CVD), including coronary heart disease and stroke (1, 2). Globally, the prevalence of CP has been estimated at 21.1% (3). In China, earlier meta-analytic evidence suggested a prevalence of 20.15%, whereas more recent modelling analyses indicate an increase to 26.27% (4, 5), suggesting a growing disease burden. CP is often asymptomatic in its early stages but is associated with adverse cardiovascular outcomes (6, 7). Therefore, detecting CP may help identify individuals at elevated CVD before the onset of overt clinical events.

Conventional single lipid parameters have long been associated with CVD risk (8, 9). However, these indicators may not fully capture the balance between pro-atherogenic and anti-atherogenic lipoproteins. In recent years, the non-high-density lipoprotein cholesterol (NHDL-C) to high-density lipoprotein cholesterol (HDL-C) ratio (NHHR) has been increasingly recognized as a comprehensive lipid indicator that integrates both pro-atherogenic and protective lipid components. Compared with single lipid parameters such as HDL-C and NHDL-C, NHHR may provide a more comprehensive and potentially superior assessment of lipid-related risk. Emerging evidence suggests that NHHR has shown favorable performance in predicting incident heart disease and cardiometabolic multimorbidity (10, 11). However, as most existing studies are cross-sectional, causal inference remains limited. Therefore, this cohort study aimed to investigate the association between NHHR and the risk of CP, providing scientific evidence for the early prevention of CP and related CVD.

## Materials and methods

### Study design and population

This study utilized data from the Dalian Health Management Cohort (DHMC) (ChiCTR2300073363), a large-scale and ongoing study initiated in 2014 based on physical examination population from the medical examination center of a third-class hospital in Dalian. Further details of the cohort design have been published elsewhere (2, 12).

A total of 9,679 adults (≥18 years) from the Dalian Health Management Cohort who underwent at least two carotid ultrasound examinations during routine health check-ups between January 2015 and December 2023 were initially identified. Participants were excluded if they had CP at the initial examination (*n* = 1,778), a self-reported history of stroke, coronary heart disease, heart failure, congenital heart disease, or atrial fibrillation (*n* = 11), malignant tumors or severe liver or kidney disease (*n* = 12), or missing key variables, including body weight, height, lipid profile, or blood glucose (*n* = 1,893). After these exclusions, 5,985 participants were included in the final analytical cohort (Supplementary Figure S1). The first ultrasound examination confirming the absence of CP was defined as baseline, and follow-up extended until the first detection of incident CP or the last available examination before December 31, 2023, whichever occurred first. Participants who did not develop CP were censored at their last follow-up visit.

### Ethics statement

This study adhered to the principles of the Declaration of Helsinki and was approved by the Ethics Committee of the Second Affiliated Hospital of Dalian Medical University (approval no. KY2025-603-01) (13). The cohort was registered in the Clinical Trial Registration System (registration no. CCC2023112102). Given the retrospective design and the use of anonymized data, the requirement for informed consent was waived (14). All patient data were handled in accordance with strict confidentiality and data protection protocols.

### Methods

Anthropometric measurements were performed with participants barefoot and wearing light clothing. Height and weight were measured using an SK-CK health examination system, with measurements recorded to the nearest 0.1 cm and 0.1 kg. Body mass index (BMI) was calculated as weight in kilograms divided by height in meters squared (kg/m^2^) (15). Blood pressure was measured in the seated position after at least 5 min of rest using an automated medical blood pressure monitor (Omron HBP-9020, Omron Corporation, Japan). Laboratory tests were conducted following standardized protocols. Venous blood samples were collected in the morning after at least 8 h of overnight fasting. Serum levels of fasting plasma glucose (FPG), total cholesterol (TC), triglycerides (TG), HDL-C, and low-density lipoprotein cholesterol (LDL-C) were measured using a Roche Cobas C 501 Chemistry Analyzer (Roche Diagnostics, Germany). White blood cell count (WBC) was analyzed using a Mindray BC-6900CRP automated hematology analyzer (Mindray, Shenzhen, China).

### Variable definitions

#### Exposure definition

NHHR was defined as the ratio of non-high-density lipoprotein cholesterol to high-density lipoprotein cholesterol, reflecting the balance between atherogenic and protective lipoproteins. NHDL-C and NHHR were calculated as follows (16, 17):


NHDL−C=TC[mmol/L]−HDL−C[mmol/L]

NHHR=(TC[mmol/L]−HDL−C[mmol/L])/HDL−C[mmol/L]

#### Outcome definition

CP was assessed using color Doppler ultrasonography of the bilateral carotid arteries, including the common carotid artery, carotid bifurcation, and internal carotid artery, performed by trained sonographers. CP was defined as an intima-media thickness (IMT) > 1.5 mm, a focal structure protruding into the arterial lumen by ≥0.5 mm, or a protrusion ≥50% of the surrounding IMT. Participants with carotid intima-media thickness (cIMT) thickening or CP were classified as having CAS (18). All examinations were performed by certified sonographers with substantial experience in vascular imaging, adhering to standardized acquisition protocols. Routine internal quality control procedures were implemented to ensure methodological consistency. Image acquisition and interpretation were conducted blinded to participants’ laboratory data.

### Covariate definitions

Demographic data, including sex, medical history, medication history, smoking and alcohol consumption history, were collected using structured questionnaires. Smoking status was assessed using a standardized questionnaire item asking participants whether they had ever smoked or were currently smoking. Responses were recorded as “yes” or “no.” Participants who answered “yes” were classified as having a history of smoking exposure, whereas those who answered “no” were classified as never smokers. Alcohol consumption was assessed using a questionnaire item asking participants whether they had ever consumed alcohol or were currently consuming alcohol. Responses were recorded as “yes” or “no.” Participants who answered “yes” were classified as having a history of alcohol consumption, whereas those who answered “no” were classified as never drinkers. Medication use was assessed based on self-reported information collected during routine health examinations. Due to variability in reporting, detailed classification of specific medication types was not consistently available; therefore, medication use was included as a binary variable (yes/no) in the analyses. Hypertension was defined as systolic blood pressure (SBP) ≥ 140 mmHg and/or diastolic blood pressure (DBP) ≥ 90 mmHg, self-reported physician-diagnosed hypertension, or use of antihypertensive medication (19). Diabetes was defined as FPG ≥ 7.0 mmol/L, 2-h oral glucose tolerance test ≥11.1 mmol/L, random glucose ≥11.1 mmol/L, self-reported physician-diagnosed diabetes, or use of hypoglycemic medication (20). Dyslipidemia was defined as meeting ≥1 of the following: TC ≥ 5.18 mmol/L, LDL-C ≥ 3.37 mmol/L, HDL-C ≤ 1.04 mmol/L, TG ≥ 1.7 mmol/L, or use of lipid-lowering medication (21).

### Statistical analysis

Continuous variables with approximately normal distributions are presented as mean ± SD and compared using one-way ANOVA; skewed variables are shown as median (P25, P75) and compared using rank-sum tests. Categorical variables are shown as counts (%) and compared using chi-square tests. Variables with more than 50% missing values were excluded from the multivariable analyses to avoid potential information bias and model instability. Other missing baseline values were supplemented, when appropriate, using the nearest available follow-up measurements obtained prior to the occurrence of CP (Supplementary Table S1). In addition, baseline characteristics were compared between included participants and those excluded due to missing key variables (Supplementary Table S2).

Kaplan–Meier curves with log-rank tests compared cumulative CP incidence across quartiles. The proportional hazards assumption was assessed using Schoenfeld residual tests, which did not indicate significant violations (NHHR: *p* = 0.4965; global test *p* = 0.1145). Cox proportional hazards models were subsequently applied to estimate hazard ratios (HRs) and 95% confidence intervals (CIs). Confounders were identified *a priori* using directed acyclic graphs (DAGs) (Supplementary Table S3; Supplementary Figure S3). Three models were fitted: Model 1 unadjusted; Model 2 adjusted for age, sex; Model 3 further adjusted for WBC, BMI, smoking, alcohol, diabetes, hypertension, dyslipidemia, and medication use. Dose–response relationships were examined using RCS (restricted cubic splines) with four knots placed at the 5th, 35th, 65th, and 95th percentiles of the NHHR distribution. An NHHR value of 2.0 was specified as the reference point. The RCS model was implemented using the rms package in R. Nonlinearity was assessed using a likelihood ratio test comparing models with and without spline terms. A two-piecewise Cox proportional hazards regression model was subsequently applied to further evaluate the threshold effect. The inflection point was determined by identifying the value corresponding to the maximum model likelihood. Subgroup analyses evaluated effect modification by sex, age, BMI, hypertension, diabetes, and dyslipidemia. The interactions between NHHR and these factors were tested using the likelihood ratio test within the framework of the fully adjusted Cox proportional hazards regression model (Model 3). Sensitivity analyses were performed to assess the robustness of the results by: (1) excluding participants who developed CP within the first year of follow-up; (2) excluding participants with baseline hypertension, diabetes, or dyslipidemia; (3) excluding participants reporting medication use at baseline; and (4) additionally adjusting for baseline cIMT in the fully adjusted model. The predictive performance of the models was evaluated using receiver operating characteristic (ROC) curve analysis. The area under the curve (AUC) with 95% confidence intervals was estimated, and comparisons between models were performed using the DeLong test.

Analyses were conducted using Stata 18.0, R 4.4.1, and DAGitty.^1^ Two-sided *p* < 0.05 was considered significant.

## Results

### Baseline characteristics

Participants were categorized into four groups according to NHHR levels: Q1 (0.48–2.14), Q2 (2.14–2.79), Q3 (2.79–3.56), and Q4 (3.56–18.18). The study included 5,985 participants (mean age: 45.10 ± 9.75 years; 58.38% male, 41.62% female). Over 11,725.34 person-years, 780 (13.03%) developed CP. The mean follow-up duration was 2.24 years (0.31–8.09), with a median of 1.92 years, and was similar across NHHR quartiles (1.77–2.01 years). Participants in higher NHHR quartiles were more likely to be male, have higher BMI, and have hypertension or diabetes, and greater medication use. They also exhibited higher SBP, DBP, FPG, TG, LDL-C, WBC, and NHDL-C levels (Table 1, *p* < 0.001). Participants with incident CP had higher NHHR levels than those without (Supplementary Table S4).

**Table 1:** Baseline characteristics of participants (2015–2023).

Variables	NHHR quartile					*p-*value
	Overall (*n* = 5,985)	Quartile 1 (*n* = 1,497)	Quartile 2 (*n* = 1,496)	Quartile 3 (*n* = 1,497)	Quartile 4 (*n* = 1,495)	
Male, *n*(%)	3,494 (58.4)	488 (32.6)	782 (52.3)	1,012 (67.6)	1,212 (81.1)	<0.001
Age, years	46 (37–52)	44 (36–50)	46 (37–52)	46 (38–52)	47 (38–53)	<0.001
BMI, kg/m^2^	24.39 (22.20–26.83)	22.13 (20.32–24.22)	24.04 (21.91–26.03)	24.93 (23.12–27.13)	26.40 (24.38–28.73)	<0.001
SBP, mmHg	125 (114–135)	120 (110–130)	124 (113–134)	126 (116–135)	129 (119–138)	<0.001
DBP, mmHg	77 (70–84)	73 (66–80)	76 (70–83)	78 (70–85)	81 (73–88)	<0.001
FPG, mmol/L	5.52 (5.23–5.89)	5.36 (5.09–5.67)	5.47 (5.21–5.81)	5.59 (5.29–5.94)	5.69 (5.39–6.18)	<0.001
TG, mmol/L	1.47 (1.06–2.04)	1.01 (0.77–1.32)	1.31 (1.02–1.64)	1.62 (1.24–2.08)	2.27 (1.71–3.12)	<0.001
TC, mmol/L	4.91 (4.36–5.53)	4.45 (3.93–4.95)	4.71 (4.23–5.26)	5.03 (4.56–5.56)	5.47 (4.94–6.08)	<0.001
HDL-C, mmol/L	1.28 (1.09–1.52)	1.63 (1.45–1.86)	1.36 (1.22–1.52)	1.21 (1.09–1.35)	1.03 (0.91–1.15)	<0.001
LDL-C, mmol/L	2.70 (2.24–3.21)	2.16 (1.84–2.46)	2.60 (2.27–2.96)	2.93 (2.59–3.31)	3.28 (2.78–3.77)	<0.001
WBC, 10^9^/L	5.78 (4.93–6.78)	5.35 (4.51–6.22)	5.65 (4.87–6.59)	5.85 (5.04–6.75)	6.37 (5.43–7.42)	<0.001
Hypertension, *n*(%)	1,274 (21.3)	189 (12.6)	293 (19.6)	344 (23.0)	448 (30.0)	<0.001
Diabetes, *n*(%)	382 (6.8)	47 (3.1)	50 (3.3)	99 (6.6)	186 (12.4)	<0.001
Dyslipidemia, *n*(%)	1853 (31.0)	63 (4.2)	183 (12.2)	477 (31.9)	1,130 (75.6)	<0.001
Smoking, *n*(%)	688 (11.5)	162 (10.8)	175 (11.7)	168 (11.2)	183 (12.2)	0.647
Alcohol, *n*(%)	1,157 (19.3)	270 (18.0)	279 (18.6)	300 (20.0)	308 (20.6)	0.252
Medication use, *n*(%)	444 (7.4)	77 (5.1)	97 (6.5)	104 (6.9)	166 (11.1)	<0.001
NHDL-C	3.58 (3.03–4.20)	2.80 (2.43–3.16)	3.36 (3.00–3.75)	3.81 (3.45–4.23)	4.45 (3.98–4.97)	<0.001
NHHR	2.79 (2.14–3.56)	1.77 (1.52–1.95)	2.46 (2.30–2.62)	3.15 (2.94–3.35)	4.17 (3.82–4.75)	<0.001
Carotid plaque, *n*(%)	780 (13.0)	129 (8.6)	163 (10.9)	234 (15.6)	254 (17.0)	<0.001

NHHR quartiles were defined as follows: Q1 (0.48–2.14), Q2 (2.14–2.79), Q3 (2.79–3.56), and Q4 (3.56–18.18). BMI, body mass index; SBP, systolic blood pressure; DBP, diastolic blood pressure; FPG, fasting plasma glucose; TG, triglycerides; LDL-C, low-density lipoprotein cholesterol; WBC, white blood cell; NHDL-C, non-high-density lipoprotein cholesterol; NHHR, non-high-density lipoprotein cholesterol to high-density lipoprotein cholesterol ratio.

### CP incidence density

CP incidence densities were 37.31, 47.07, 70.25, and 80.89 per 1,000 person-years for NHHR quartiles Q1-Q4, respectively, with an overall rate of 66.52 per 1,000 person-years. Incidence was higher in men and in participants aged ≥45 years. Stratified analyses demonstrated a higher incidence of CP among participants with diabetes, hypertension, or dyslipidemia, whereas no significant association was observed when stratified by BMI (Supplementary Table S5).

### Association between NHHR quartiles and CP risk

**Figure 1:**
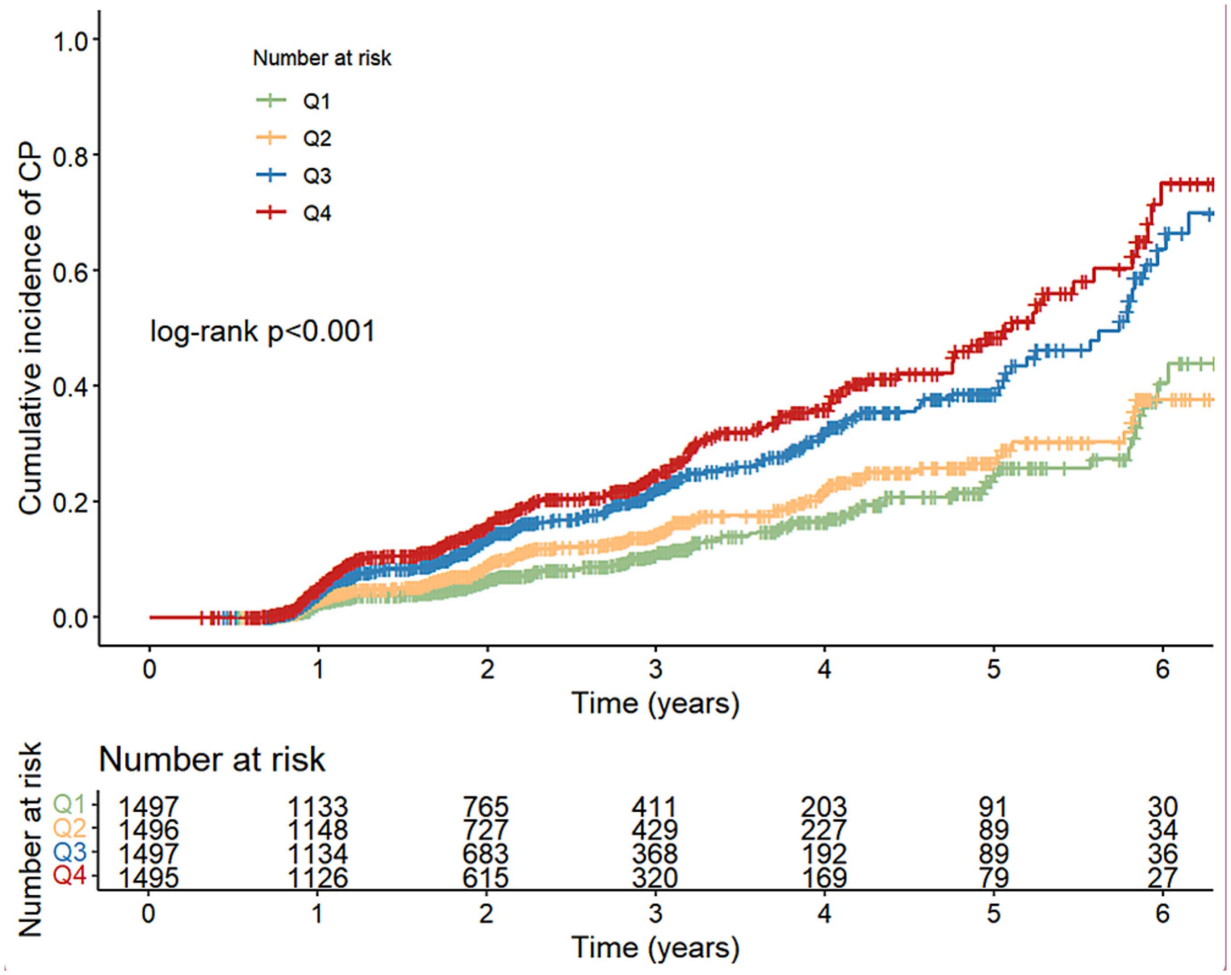
Kaplan–Meier curves of cumulative CP incidence by NHHR quartiles. CP, carotid plaque; NHHR, non-high-density lipoprotein cholesterol to high-density lipoprotein cholesterol ratio.

Kaplan–Meier curve analysis showed that the cumulative incidence of CP differed significantly across NHHR quartiles (*p* < 0.001). The incidence increased progressively from Q1 to Q4, with the highest incidence observed in Q4 and the lowest in Q1 (Figure 1). In the multivariable Cox proportional hazards model (Table 2), participants in Q4 had a 64% higher risk of CP compared with those in Q1 (HR = 1.64, 95% CI: 1.26–2.14, *p* < 0.001). A significant dose–response relationship was observed across increasing NHHR quartiles (P for trend < 0.001). When NHHR was treated as a continuous variable, the association remained statistically significant (HR = 1.11, 95% CI: 1.04–1.18, *p* = 0.001). ROC analysis showed that adding NHHR slightly improved model discrimination, with the AUC increasing from 0.738 to 0.741 (DeLong test *p* = 0.050) (Supplementary Figure S4).

**Table 2:** Cox regression models for CP risk by baseline NHHR quartiles.

NHHR quartile	Model 1		Model 2		Model 3	
	HR (95%CI)	*p* value	HR (95%CI)	*p* value	HR (95%CI)	*p* value
Quartile 1	Reference		Reference		Reference	
Quartile 2	1.26 (0.99–1.58)	0.053	1.04 (0.82–1.31)	0.764	1.02 (0.81–1.30)	0.852
Quartile 3	1.90 (1.53–2.35)	<0.001	1.54 (1.25–1.91)	<0.001	1.53 (1.22–1.93)	<0.001
Quartile 4	2.24 (1.81–2.77)	<0.001	1.72 (1.39–2.14)	<0.001	1.64 (1.26–2.14)	<0.001
Continuous NHHR	1.21 (1.15–1.27)	<0.001	1.15 (1.09–1.21)	<0.001	1.11 (1.04–1.18)	0.001
P for trend	/	<0.001	/	<0.001	/	<0.001

Model 1 was unadjusted. Model 2 was adjusted for age and sex. Model 3 was further adjusted for BMI, WBC, smoking, alcohol, hypertension, diabetes, dyslipidemia, and medication use. BMI, body mass index; WBC, white blood cell; NHHR, represents the ratio of non-high-density lipoprotein cholesterol to high-density lipoprotein cholesterol; CP, carotid plaque.

### Restricted cubic spline and threshold-effect analyses

**Figure 2:**
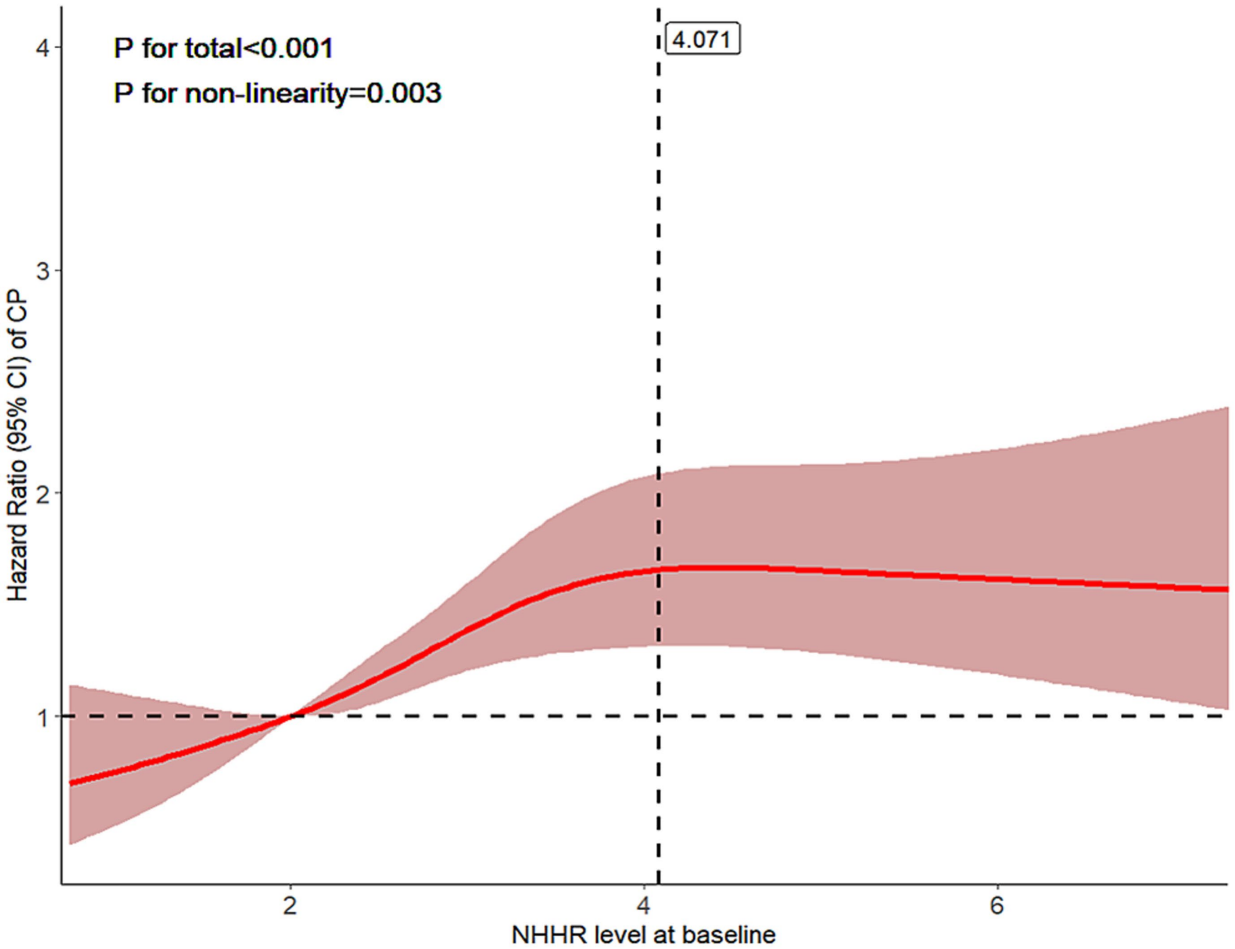
Association between NHHR and CP risk assessed using restricted cubic spline analysis. RCS with four knots were used to explore the potential nonlinear association, with knots placed at the 5th, 35th, 65th, and 95th percentiles of the NHHR distribution. Model 3 was adjusted for age, sex, BMI, WBC, smoking, alcohol, hypertension, diabetes, dyslipidemia, and medication use. CP, carotid plaque; NHHR, non-high-density lipoprotein cholesterol to high-density lipoprotein cholesterol ratio.

The RCS analysis demonstrated a nonlinear association between NHHR and CP risk. With the continuous increase of NHHR, CP risk gradually increases (Supplementary Figure S5). After adjusting the covariates, the trend of the results is consistent (Figure 2). Threshold effect analysis further supported the nonlinear association between NHHR and CP risk (Supplementary Table S6). In the standard Cox proportional hazards model (linear term), NHHR was significantly associated with CP risk (HR = 1.111, 95%CI: 1.041–1.186, *p* = 0.002). In the two-piecewise Cox proportional hazards model, NHHR was positively associated with CP risk below 4.071 (HR = 1.316, 95%CI: 1.173–1.476, *p* < 0.001), whereas no significant association was observed above 4.071. The likelihood ratio test results (*p* < 0.001) further confirmed the existence of this threshold effect.

### Subgroup analysis of NHHR and CP risk

Subgroup analyses were performed to assess potential effect modification by sex, age, BMI, hypertension, diabetes, and dyslipidemia status. Statistically significant interactions (assessed by likelihood ratio test) were observed for age, sex, and hypertension (all P for interaction < 0.05). This indicates that the positive association between NHHR and CP risk was significantly stronger among younger participants (<45 years) compared to their older counterparts (≥45 years), in females compared to males, and in normotensive individuals compared to those with hypertension. In contrast, no significant interaction was observed for BMI, diabetes status, or dyslipidemia (all P for interaction > 0.05), suggesting that the association was consistent across these subgroups (Table 3; Supplementary Figure S6).

**Table 3:** Subgroup analysis of NHHR as a continuous variable.

Variables	Model 3 HR (95%CI)	*p* value	*P* for interaction
Age, years			<0.001
<45	1.31(1.17–1.47)	<0.001	
≥45	1.04(0.96–1.12)	0.380	
Sex			<0.001
Female	1.18(1.01–1.39)	0.040	
Male	1.10(1.02–1.18)	0.012	
BMI (kg/m^2^)			0.987
<24	1.18(1.04–1.35)	0.012	
≥24	1.09(1.01–1.18)	0.030	
Hypertension			0.021
No	1.16(1.06–1.26)	0.001	
Yes	1.06(0.96–1.17)	0.287	
Diabetes			0.529
No	1.12(1.04–1.21)	0.002	
Yes	1.06(0.91–1.23)	0.435	
Dyslipidemia			0.468
No	1.14(1.07–1.21)	<0.001	
Yes	1.06(0.93–1.20)	0.390	

Model 3 was adjusted for age, sex, BMI, WBC, smoking, alcohol, hypertension, diabetes, dyslipidemia, and medication use. BMI, body mass index.

### Sensitivity analysis

Sensitivity analyses were performed to assess the robustness of the findings, which remained consistent with the primary analysis (Supplementary Table S7). The association between NHHR and CP persisted after excluding participants who developed CP within the first year of follow-up (HR = 1.76, 95% CI: 1.30–2.39, *p* < 0.001), those with baseline hypertension, diabetes, or dyslipidemia (HR = 2.39, 95% CI: 1.54–3.71, *p* < 0.001), and those reporting medication use at baseline (HR = 1.79, 95% CI: 1.33–2.42, *p* < 0.001). Further adjustment for baseline cIMT did not materially change the results, with the highest NHHR quartile still associated with an increased risk of CP (HR = 1.67, 95% CI: 1.28–2.19, *p* < 0.001) (Supplementary Table S8).

## Discussion

In this cohort, elevated NHHR was independently associated with higher CP risk. In fully adjusted models, participants in Q4 had a 1.64-fold higher risk of CP compared with those in Q1. RCS revealed a nonlinear positive association with an inflection at NHHR = 4.071; below this point, risk rose significantly with NHHR, while above this point, the NHHR-related risk of new-onset CP remained relatively stable. Subgroup analyses identified significant interactions of NHHR with age, sex, and hypertension status. Multiple sensitivity analyses confirmed robustness, supporting NHHR as an independent risk marker of CP.

NHHR, as a novel composite biomarker for health risk assessment, integrates levels of pro-atherogenic lipoproteins and protective HDL-C, making it superior to traditional single lipid indicators in evaluating atherosclerosis risk (22, 23). Recent research highlights NHHR’s enhanced performance in predicting cardiovascular events (24, 25). For instance, the Atherosclerosis Risk in Communities (ARIC) study demonstrated an independent association between NHHR and lipid cores in carotid plaques (26). Additional evidence corroborates NHHR’s close links to carotid atherosclerosis progression and plaque stability (27, 28). Our findings align with these, showing that Q4 participants had a 1.64-fold higher CP risk than Q1, indicating a progressive increase in CP prevalence with rising NHHR levels (28). This is consistent with prior studies, such as that by Qin et al. (29), which reported a stepwise increase in cIMT across NHHR quartiles, collectively underscoring the strong connection between NHHR and CP risk.

The restricted cubic spline analysis revealed a nonlinear positive correlation between NHHR and CP risk, with an identified inflection point at 4.071. Above this threshold, the risk increase remained relatively stable. This pattern may be explained by the exclusion of participants with pre-existing CP at baseline, and the limited sample size at higher NHHR levels. Therefore, this finding requires validation in larger studies. In addition, the potential occurrence of cardiovascular events during follow-up may also influence the observed association, which warrants further investigation. Similar nonlinear relationships have also been reported between NHHR and other adverse metabolic outcomes (11, 30). Notably, the threshold of NHHR varies across clinical outcomes: inflection points for all-cause and cardiovascular mortality in adults with diabetes or prediabetes were 2.72 and 2.83 (31), respectively, whereas a threshold of 6.28 was identified for cardiovascular outcomes in patients with type 2 diabetes (32). Previous subgroup analyses have indicated significant associations between NHHR and CP stability across strata of sex, age, hypertension, status (28). Our findings further demonstrated that NHHR was associated with higher hazard ratios in individuals aged <45 years, females, and those without hypertension. Possible explanations include: First, the cohort’s exclusion of high-risk individuals at baseline may amplify NHHR’s early warning value in relatively healthy populations. Second, younger adults often exhibit better lipid metabolic compensation, rendering early-stage atherosclerosis more sensitive to mild dyslipidemia (33, 34), where inflammation and lipid abnormalities synergistically drive progression (35). Third, the stronger NHHR–CP association observed in females in our sex-stratified analysis may be associated with sex hormone-related differences in lipid metabolism, including the regulatory effects of estrogen on HDL functionality and LDL phenotypes (36, 37). This echoes previous findings of a more pronounced NHHR-CP association in women (38). Finally, hypertension independently accelerates atherosclerosis, potentially diminishing NHHR’s relative predictive power in hypertensive individuals (39), leading to weaker risk differentiation.

The underlying mechanisms by which NHHR promotes CP formation likely involve multiple pathways. Elevated NHHR signifies an increased burden of atherogenic lipoproteins coupled with diminished protective HDL-C function, collectively fostering endothelial dysfunction (40). Studies have linked higher NHHR to reduced nitric oxide bioavailability and elevated endothelin-1 (ET-1) levels (41). Inflammation plays a pivotal role in this association, with NHHR elevation correlating to increased levels of inflammatory markers such as high-sensitivity C-reactive protein (hs-CRP), interleukin-6 (IL-6), and tumor necrosis factor-alpha (TNF-*α*) (42, 43). Oxidative stress represents another key mechanism, as high NHHR level is associated with elevated oxidized low-density lipoprotein (ox-LDL) and reduced antioxidant enzyme activity (44). Furthermore, NHHR’s interconnections with metabolic factors like obesity, hyperglycemia, and hypertension may exacerbate atherosclerosis progression (45). These mechanisms indirectly bolster our conclusions, affirming NHHR’s clinical utility in predicting CP risk.

The strengths of this study include its use of large-scale cohort data to substantiate the association between NHHR and CP, reducing the likelihood of reverse causation. Comprehensive control for confounding was achieved through directed acyclic graphs, multiple modelling approaches, subgroup analyses, and sensitivity tests, all of which support the robustness of the findings. Notably, the heightened predictive efficacy of NHHR in younger, female, and non-hypertensive subgroups provides evidence-based guidance for precision screening in specific populations. During a mean follow-up of 2.24 years, 13.03% of participants developed CP. When both baseline cases (*n* = 1,778) and incident cases (*n* = 780) were considered within the initially screened population (*n* = 9,679), the overall proportion of CP was broadly comparable in magnitude to the approximately 26% prevalence reported in recent studies in China, supporting the representativeness of our study population, although such comparisons should be interpreted cautiously.

However, several limitations should be considered. Firstly, although NHHR was significantly associated with CP risk, its incremental predictive value was modest, and residual confounding from unmeasured or unknown factors cannot be entirely excluded, as some baseline variables were missing and medication use was not fully captured. Secondly, the study population was exclusively Chinese and derived from a routine health examination cohort, which may limit the generalizability of the findings to other populations and necessitates external validation in diverse settings. Thirdly, CP was assessed using ultrasonography, which is operator-dependent and may be subject to interobserver bias despite standardized procedures. In addition, the lack of quantitative assessment of plaque morphology, stability, or phenotypic characteristics precludes a more detailed evaluation of the relationship between NHHR and plaque features. Future prospective studies with more comprehensive data collection and repeated measurements are warranted.

## Conclusion

NHHR is independently associated with the risk of CP. As an inexpensive and readily available biomarker derived from routine lipid testing, NHHR may assist in identifying individuals at elevated risk of CP. Further prospective studies are warranted to confirm its clinical relevance.

## Data Availability

The raw data supporting the conclusions of this article will be made available by the authors, without undue reservation.

## Footnotes

1
http://www.dagitty.net/develop/dags.html

## Glossary

ANOVA: analysis of variance
BMI: body mass index
CAS: carotid atherosclerosis
CI: confidence intervals
CVD: cardiovascular disease
DAGs: directed acyclic graphs
DBP: diastolic blood pressure
ET-1: endothelin-1
FPG: fasting plasma glucose
HDL-C: high-density lipoprotein cholesterol
HR: hazard ratios
hs-CRP: high-sensitivity C-reactive protein
IL-6: interleukin-6
IMT: intima-media thickness
LDL-C: low-density lipoprotein cholesterol
NHHR: non-high-density lipoprotein cholesterol to high-density lipoprotein cholesterol ratio
NHDL-C: non-high-density lipoprotein cholesterol
ox-LDL: oxidized low-density lipoprotein
RCS: restricted cubic splines
ROC: receiver operating characteristic
SBP: systolic blood pressure
TC: total cholesterol
TG: triglycerides
TNF-*α*: tumor necrosis factor-alpha
WBC: white blood cell count
cIMT: carotid intima-media thickness
CP: carotid plaque
