# Supplementary material for: Association between the non-high-density lipoprotein cholesterol to high-density lipoprotein cholesterol ratio and carotid plaque: a retrospective cohort study
Source: Front Nutr. 2026 Apr 28;13:1792724. doi: 10.3389/fnut.2026.1792724 (PMC13160848; doi:10.3389/fnut.2026.1792724)
Supplement: Supplementary file 1 [file Data_Sheet_1.pdf]

Supplementary Materials

Table S1. Comparison of baseline characteristics before and after imputation

Table S2. Baseline comparison between included participants and participants excluded due to missing key variables

Table S3. Single factor Cox regression analysis with CP as the outcome

Table S4. Baseline characteristics based on follow-up outcomes with or without CP

Table S5. Comparison of CP incidence density and incidence rate in different subgroups

Table S6 Threshold effect of NHHR on CP risk based on a two-piecewise Cox regression model

Table S7. Sensitivity analysis 1-3 between NHHR and CP

Table S8. Sensitivity analysis4 between NHHR and carotid plaque

Figure S1. Flowchart of Participant Inclusion and Exclusion

Figure S2. Proportional hazard hypothesis test for association between NHHR and CP

Figure S3. Directed acyclic graph

Figure S4. ROC curves for traditional and NHHR-based models predicting CP.

Figure S5. Restricted Cubic Spline Curve of NHHR and CP Risk

Figure S6. Subgroup analysis chart between NHHR and CP

Table S1. Comparison of baseline characteristics before and after imputation

Variable	Before imputation (n=5985)	After imputation (n=5985)	SMD
Smoking, n (%)	595 (11.6)	688 (11.5)	0.002
Alcohol, n(%)	1032 (19.6)	1157 (19.3)	0.007

Only variables with missing values that required imputation were included in this comparison. SMD < 0.1 indicates negligible differences between datasets.

Table S2. Baseline comparison between included participants and participants excluded due to missing key variables

**Variable**	**Excluded (n = 673)**	**Included (n = 5985)**	***P* value**
Male, n(%)	229 (34.0)	3494 (58.4)	<0.001
Age, years	49(44-54)	46(37-52)	<0.001
BMI, kg/m2	24.61(22.66-26.50)	24.39(22.20-26.84)	0.426
SBP, mmHg	126(116-137)	125(114.00-135)	0.005
DBP, mmHg	78(71-86)	77(70.00-84.00)	0.002
FPG,mmol/L	5.66(5.35-6.08)	5.52(5.23-5.89)	<0.001
WBC, 109/L	5.74(4.85-6.62)	5.78(4.93-6.78)	0.094
TG, mmol/L	1.51(1.01-2.07)	1.47(1.06-2.04)	0.943
LDL-C, mmol/L	2.99(2.44-3.56)	2.70(2.24-3.21)	<0.001
Hypertension, n(%)	188 (27.9)	1274 (21.3)	<0.001
Diabetes, n(%)	61 (9.1)	382 (6.4)	0.010
Dyslipidemia, n(%)	267 (39.7)	1853 (31.0)	<0.001
Smoking, n(%)	22 (3.6)	688 (11.5)	<0.001
Alcohol, n(%)	13 (2.1)	1157 (19.3)	<0.001
Medication use, n (%)	103 (15.3)	444 (7.4)	<0.001

Abbreviations: BMI, body mass index; SBP, systolic blood pressure; DBP, diastolic blood pressure; FPG, fasting plasma glucose; TG, triglycerides; LDL-C, low-density lipoprotein cholesterol; WBC, white blood cell.

Table S3. Single factor Cox regression analysis with CP as the outcome

**Variables**	***HR* (95% *CI*)**	***P* value**
Male, n(%)	1.957(1.670-2.295)	<0.001
Age, years	1.070(1.063-1.077)	<0.001
BMI(kg/m^2)	1.056(1.039-1.073)	<0.001
SBP(mmHg)	1.023(1.019-1.027)	<0.001
DBP(mmHg)	1.031(1.025-1.037)	<0.001
FPG (mmol/L)	1.152(1.109-1.196)	<0.001
TG(mmol/L)	1.147(1.090-1.207)	<0.001
TC(mmol/L)	1.290(1.202-1.386)	<0.001
HDL-C(mmol/L)	0.596(0.479-0.741)	<0.001
LDL-C(mmol/L)	1.531(1.395-1.680)	<0.001
WBC(10^9/L)	1.062(1.017-1.110)	0.007
Hypertension, n(%)	2.260(1.951-2.618)	<0.001
Diabetes, n(%)	2.092(1.677-2.609)	<0.001
Dyslipidemia, n(%)	1.484(1.285-1.714)	<0.001
Smoking, n(%)	1.578(0.946-2.634)	0.081
Alcohol, n(%)	1.120(0.755-1.906)	0.441
Medication use, n (%)	2.671(2.181-3.272)	<0.001
NHDL-C	1.364(1.266-1.469)	<0.001
NHHR	1.209(1.161-1.259)	<0.001

Abbreviations: BMI, body mass index; SBP, systolic blood pressure; DBP, diastolic blood pressure; FPG, fasting plasma glucose; TG, triglycerides; TC, total cholesterol; HDL-C, high-density lipoprotein cholesterol; LDL-C, low-density lipoprotein cholesterol; WBC, white blood cell; NHDL-C, non-high-density lipoprotein cholesterol; NHHR, non-high-density lipoprotein cholesterol to high-density lipoprotein cholesterol ratio; CP, carotid plaque.

Table S4. Baseline characteristics based on follow-up outcomes with or without CP

Variables	Overall (n=5985)	No carotid plaque (n=5205)	New carotid plaque (n=780)	*P* value
Male, n(%)	3494(58.38)	2922 (56.1)	572 (73.3)	<0.001
Age, years	46(37-52)	45(37-51)	51(46-57)	<0.001
BMI, kg/m^2	24.39(22.20-26.83)	24.28(22.04-26.71)	25.28(23.24-27.40)	<0.001
SBP, mmHg	125(114-135)	124(114-134)	131(120-140)	<0.001
DBP, mmHg	77(70-84)	76(69-84)	82(73-89)	<0.001
FPG, mmol/L	5.52(5.23-5.89)	5.50(5.22-5.86)	5.69(5.38-6.14)	<0.001
TG, mmol/L	1.47(1.06-2.04)	1.44(1.04-2.01)	1.67(1.24-2.33)	<0.001
TC, mmol/L	4.91(4.36-5.53)	4.88(4.33-5.51)	5.12(4.55-5.71)	<0.001
HDL-C, mmol/L	1.28(1.09-1.52)	1.29(1.09-1.53)	1.24(1.07-1.46)	<0.001
LDL-C, mmol/L	2.70(2.24-3.21)	2.68(2.22-3.19)	2.88(2.45-3.32)	<0.001
WBC, 10^9/L	5.78(4.93-6.78)	5.76(4.92-6.75)	5.94(4.99-6.95)	0.002
Hypertension, n(%)	1274(21.3)	991(19.0)	283(36.3)	<0.001
Diabetes, n(%)	382(6.8)	289(5.6)	93(11.9)	<0.001
Dyslipidemia, n(%)	1853(31.0)	1550(29.8)	303(38.8)	<0.001
Smoking, n(%)	688(11.5)	595(11.4)	93(11.9)	0.733
Alcohol, n(%)	1157(19.3)	986(18.9)	171(21.9)	0.055
Medication use, n (%)	444(7.4)	331(6.4)	113(14.5)	<0.001
NHDL-C	3.58(3.03-4.20)	3.54(3-4.16)	3.85(3.28-4.40)	<0.001
NHHR	2.79(2.14-3.56)	2.74(2.11-3.52)	3.05(2.42-3.84)	<0.001

Abbreviations: BMI, body mass index; SBP, systolic blood pressure; DBP, diastolic blood pressure; FPG, fasting plasma glucose; TG, triglycerides; TC, total cholesterol ; HDL-C, high-density lipoprotein cholesterol; LDL-C, low-density lipoprotein cholesterol; WBC, white blood cell; NHDL-C, non-high-density lipoprotein cholesterol; NHHR, non-high-density lipoprotein cholesterol to high-density lipoprotein cholesterol ratio; CP, carotid plaque.

Table S5. Comparison of CP incidence density and incidence rate in different subgroups

Categories	Event/Total	Incidence rate (%)	Total person-years	Incidence density (/1000 person-years)	χ^2 value	*P* value
NHHR					75.16	<0.001
Q1	129/1497	8.62	3457.55	37.31		
Q2	163/1496	10.89	3462.72	47.07		
Q3	234/1497	15.63	3330.82	70.25		
Q4	254/1495	16.99	3139.96	80.89		
Sex					71.03	<0.001
Male	572/3494	16.37	7773.20	73.59		
Female	208/2491	8.35	5617.85	37.02		
Age(years)					247.00	<0.001
<45	147/2726	5.39	6225.10	23.61		
≥45	633/3259	19.42	7165.95	88.33		
Diabetes					46.72	<0.001
Yes	93/382	24.08	827.80	112.34		
No	687/5603	12.26	12563.25	54.68		
Hypertension					126.56	<0.001
Yes	283/1274	22.21	2724.76	103.86		
No	497/4711	10.55	10666.29	46.60		
Dyslipidemia					29.28	<0.001
Yes	303/1853	16.35	4043.81	74.93		
No	477/4132	11.54	9347.24	51.03		
BMI					2.83	0.093
<24	146/992	14.71	2191.91	66.61		
≥24	634/4993	12.70	11199.14	56.61		
cIMT					97.69	<0.001
Yes	25/37	67.56	52.20	478.93		
No	755/5948	12.69	13338.85	56.60		
Total	780/5985	13.03	11725.34	66.52		

Incidence density = Event/Total person-years *1000

Abbreviations: NHHR, non-high-density lipoprotein cholesterol to high-density; BMI, body mass index; CP, carotid plaque; cIMT, carotid intima-media thickness.

Table S6. Threshold effect of NHHR on CP risk based on a two-piecewise Cox regression model

Variables	Effect Size, 95% CI	*P* value
Model 1: Single linear Cox model	1.111 (1.041-1.186)	0.002
Model 2: Two-piecewise Cox model		
Inflection point	4.071	
<4.071	1.316 (1.173-1.476)	<0.001
≥4.071	0.953 (0.838-1.083)	0.459
P for likelihood ratio test		<0.001

Model 1 represents the single linear Cox model; Model 2 represents the two-piecewise Cox model with an inflection point at 4.071. Model2 was adjusted for age, sex, BMI, WBC, smoking, alcohol, hypertension, diabetes, dyslipidemia, and medication use.

Abbreviations: Effect sizes are presented as hazard ratios with 95% confidence intervals (CI); NHHR, non-high-density lipoprotein cholesterol to high-density lipoprotein cholesterol ratio; CP, carotid plaque.

Table S7. Sensitivity analysis1-3 between NHHR and CP.

NHHR quartile	Model 1		Model 2		Model 3	
	**HR (95%CI)**	***P* value**	**HR (95%CI)**	***P* value**	**HR (95%CI)**	***P* value**
**Analysis 1**						
Quartile 1	Reference		Reference		Reference	
Quartile 2	1.26(0.99-1.64)	0.087	1.03(0.78-1.35)	0.840	1.02(0.78-1.34)	0.879
Quartile 3	1.98(1.51-2.53)	<0.001	1.60(1.25-2.05)	<0.001	1.62(1.25-2.10)	0.001
Quartile 4	2.31(1.82-2.95)	<0.001	1.78(1.38-2.28)	<0.001	1.76(1.30-2.39)	<0.001
Continuous NHHR	1.21(1.16-1.27)	<0.001	1.15(1.09-1.22)	<0.001	1.11(1.03-1.20)	0.004
**Analysis 2**						
Quartile 1	Reference		Reference		Reference	
Quartile 2	1.45(1.06-1.97)	0.020	1.30(0.95-1.78)	0.102	1.29(0.94-1.78)	0.117
Quartile 3	2.31(1.70-3.13)	<0.001	1.90(1.39-2.60)	<0.001	1.88(1.35-2.62)	<0.001
Quartile 4	3.13(2.13-4.59)	<0.001	2.49(1.64-3.78)	<0.001	2.39(1.54-3.71)	<0.001
Continuous NHHR	1.73(1.51-2.00)	<0.001	1.56(1.32-1.85)	<0.001	1.55(1.29-1.85)	<0.001
**Analysis 3**						
Quartile 1	Reference		Reference		Reference	
Quartile 2	1.36(1.06-1.75)	0.018	1.11(0.86-1.44)	0.417	1.08(0.83-1.41)	0.572
Quartile 3	2.11(1.67-2.67)	<0.001	1.68(1.33-2.13)	<0.001	1.63(1.26-2.10)	<0.001
Quartile 4	2.46(1.94-3.10)	<0.001	1.89(1.49-2.41)	<0.001	1.79(1.33-2.42)	<0.001
Continuous NHHR	1.23(1.17-1.29)	<0.001	1.17(1.11-1.24)	<0.001	1.13(1.06-1.22)	0.001

Model 1 was unadjusted. Model 2 was adjusted for age and sex. Model 3 was further adjusted for BMI, WBC, smoking, alcohol, hypertension, diabetes, dyslipidemia, and medication use.

Analysis 1 excluding CP within the first year (n=5,796) to reduce reverse causality; Analysis 2 excluding baseline hypertension, diabetes, or dyslipidemia (n=3,321); Analysis 3 excluding participants who reported medication use at baseline (n = 5,541).

Abbreviations: BMI, body mass index; WBC, white blood cell; CP, carotid plaque; NHHR, non-high-density lipoprotein cholesterol to high-density lipoprotein cholesterol ratio.

Table S8. Sensitivity analysis4 between NHHR and carotid plaque

NHHR quartile	Model 3	
	HR (95%CI)	P value
**Analysis 4**		
Quartile 1	Reference	
Quartile 2	1.04(0.82-1.32)	0.727
Quartile 3	1.52(1.21-1.92)	<0.001
Quartile 4	1.67(1.28-2.19)	<0.001
Continuous NHHR	1.12(1.05-1.19)	<0.001

Model 3 adjusted for age, sex, cIMT, BMI, WBC, smoking, alcohol, hypertension, diabetes, dyslipidemia, and medication use.

This sensitivity analysis additionally adjusted for baseline carotid intima-media thickness (cIMT) to assess its potential impact on the association between NHHR and incident carotid plaque. The results remained materially unchanged, supporting the robustness of the main findings.

NHHR, non-high-density lipoprotein cholesterol to high-density lipoprotein cholesterol ratio

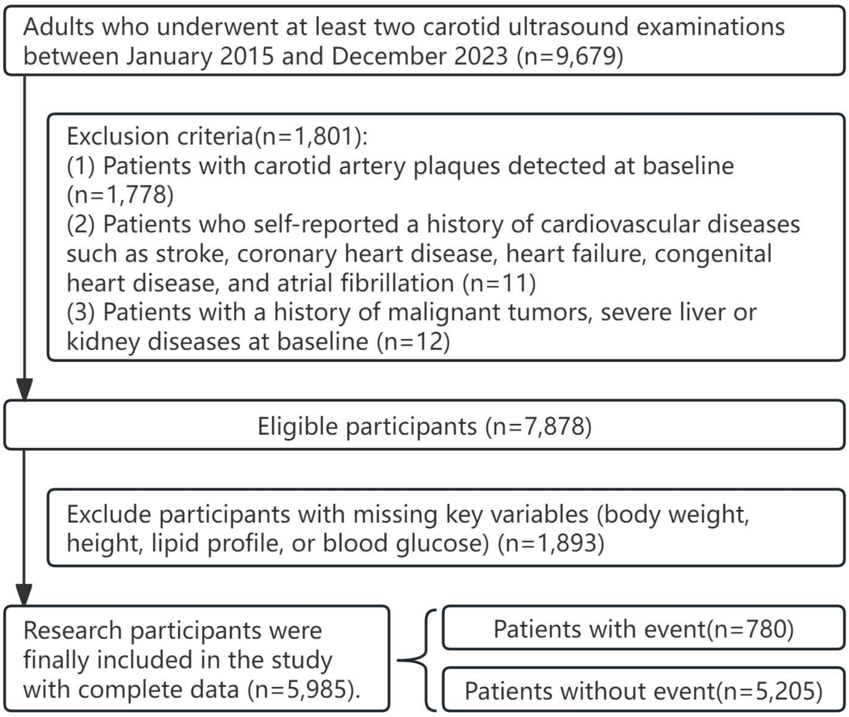

Figure S1. Flowchart of Participant Inclusion and Exclusion

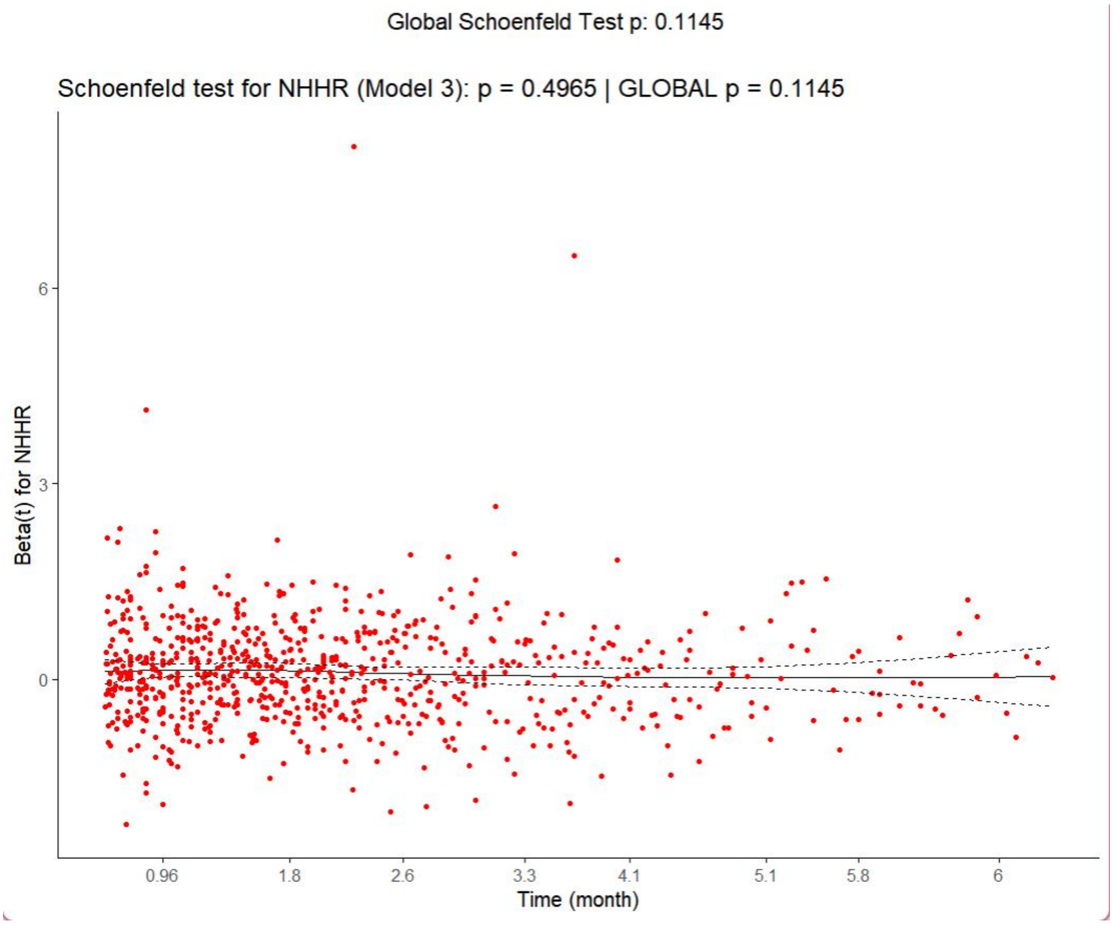

Figure S2. Proportional hazard hypothesis test for association between NHHR and CP

The Schoenfeld residuals test indicated no significant violation of the proportional hazards assumption for NHHR (p = 0.4965; global p = 0.1145).

Abbreviations: NHHR, non-high-density lipoprotein cholesterol to high-density lipoprotein cholesterol ratio; CP, carotid plaque.

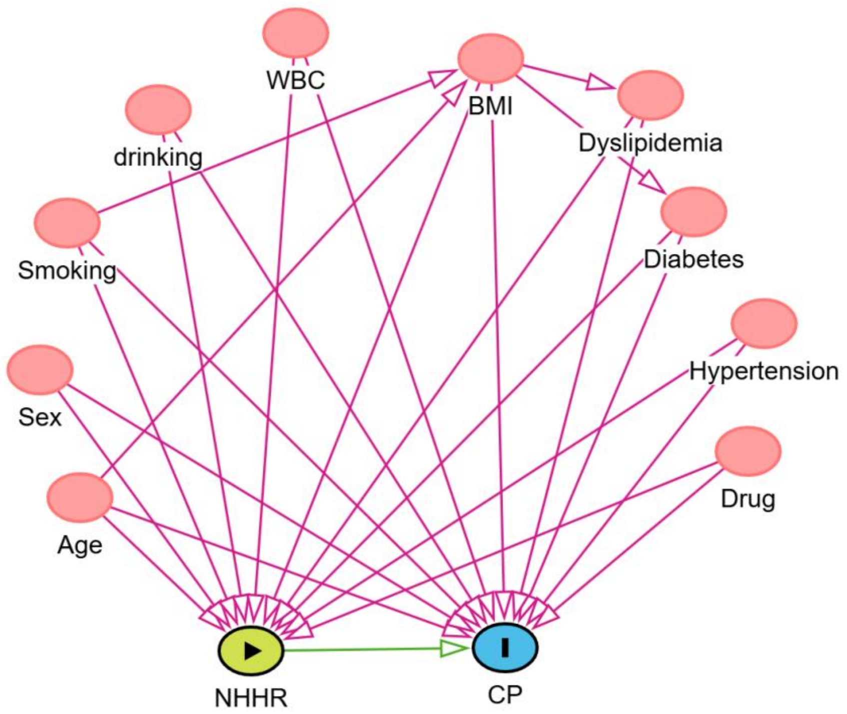

Figure S3. Directed acyclic graph

DAG illustrating the relationships among smoking, hypertension, dyslipidemia, diabetes, age, sex, and drug use and their potential effects on NHHR and CP. Green circles represent the exposure (NHHR), blue circles represent the outcome (CP), and pink circles indicate confounding factors requiring adjustment. Arrows denote direct associations, with green arrows highlighting pathways indicating effects on NHHR and CP.

Abbreviations: BMI, body mass index; WBC, white blood cell; CP, carotid plaque; NHHR, non-high-density lipoprotein cholesterol to high-density lipoprotein cholesterol ratio;

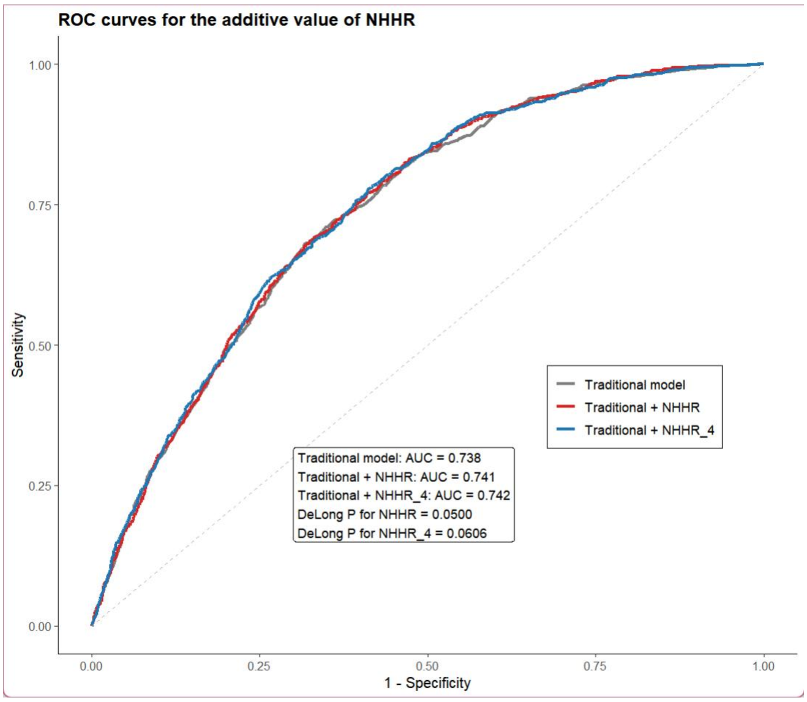

Figure S4. ROC curves for traditional and NHHR-based models predicting CP.

The area under the curve (AUC) was 0.738 for the traditional model, 0.741 after adding continuous NHHR, and 0.742 after adding NHHR quartile categories (NHHR_4). The improvement in discrimination was borderline significant for continuous NHHR (DeLong test, P = 0.0500), whereas the increment for NHHR quartile categories was not statistically significant (DeLong test, P = 0.0606). The traditional model included age, sex, smoking, alcohol, hypertension, and diabetes. Abbreviations: ROC, receiver operating characteristic; NHHR, non-high-density lipoprotein cholesterol to high-density lipoprotein cholesterol ratio.

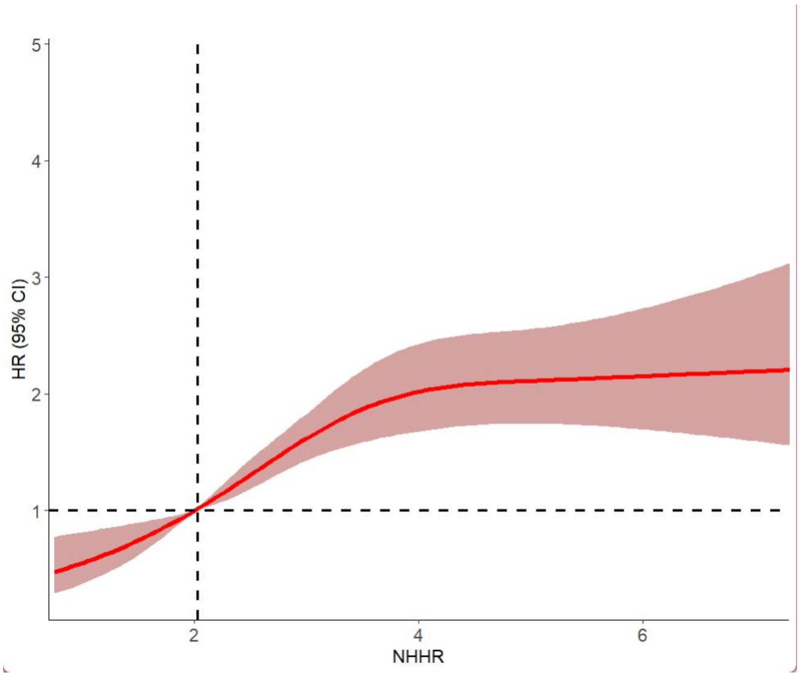

Figure S5. Restricted Cubic Spline Curve of NHHR and CP Risk

RCS with four knots were used to explore the potential nonlinear association, with knots placed at the 5th, 35th, 65th, and 95th percentiles of the NHHR distribution. This model has not adjusted any variables.

Abbreviations: CP represents carotid plaque; NHHR, non-high-density lipoprotein cholesterol to high-density lipoprotein cholesterol ratio.

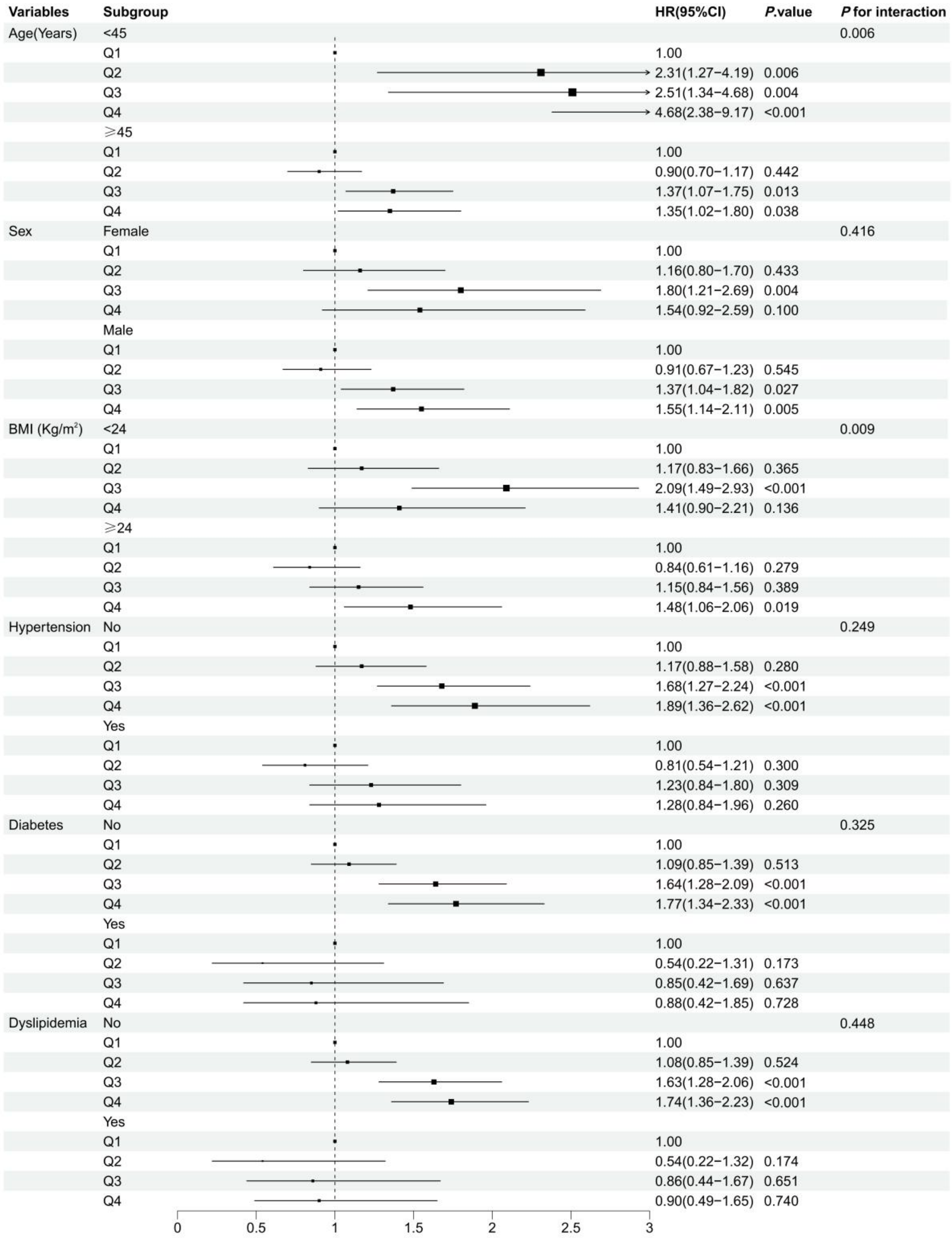

Figure S6. Subgroup analysis chart between NHHR and CP

Model 3 was adjusted for age, sex, BMI, WBC, smoking, alcohol, hypertension, diabetes, dyslipidemia, and medication use.

Abbreviations: BMI, body mass index; WBC, white blood cell;
